# ECDC’s latest publications

**DOI:** 10.2807/1560-7917.ES.2019.24.39.1909262

**Published:** 2019-09-26

**Authors:** Eurosurveillance editorial team

**Affiliations:** 1 European Centre for Disease Prevention and Control (ECDC), Stockholm, Sweden

**Keywords:** infectious diseases, public health, Europe

 

**Rapid risk and outbreak assessments**

 

Ebola virus disease outbreak in North Kivu and Ituri Provinces, Democratic Republic of the Congo - sixth update

Date of publication: 7 August 2019

https://ecdc.europa.eu/en/publications-data/rapid-risk-assessment-ebola-virus-disease-outbreak-north-kivu-and-ituri-1

**

Ebola virus disease outbreak in North Kivu and Ituri Provinces, Democratic Republic of the Congo – fifth update

Date of publication: 19 July 2019

https://ecdc.europa.eu/en/publications-data/RRA-ebola-virus-disease-outbreak-DRC-fifth-update

 

Public health risks related to communicable diseases during the Hajj 2019, Saudi Arabia, 9–14 August 2019

Date of publication: 2 July 2019

https://ecdc.europa.eu/en/publications-data/rapid-risk-assessment-public-health-risks-related-communicable-diseases-during-1

 

**Technical reports**

 

Options for national testing and surveillance for hepatitis E virus in the EU/EEA - Operational guidance

Date of publication: 5 September 2019

https://ecdc.europa.eu/en/publications-data/options-national-testing-and-surveillance-hepatitis-e-virus-eueea-operational

 

Developing a national strategy for the prevention and control of sexually transmitted infections

Date of publication: 4 September 2019

https://ecdc.europa.eu/en/publications-data/developing-national-strategy-prevention-and-control-sexually-transmitted

 

Euro-GASP external quality assessment scheme for *Neisseria gonorrhoeae* antimicrobial susceptibility testing

Date of publication: 3 September 2019

https://ecdc.europa.eu/en/publications-data/euro-gasp-external-quality-assessment-scheme-neisseria-gonorrhoeae-antimicrobial

 

EMIS-2017 – The European Men-Who-Have-Sex-With-Men Internet Survey

Date of publication: 29 August 2019

https://ecdc.europa.eu/en/publications-data/emis-2017-european-men-who-have-sex-men-internet-survey

 

Sixth external quality assessment scheme for *Listeria monocytogenes* typing

Date of publication: 26 August 2019

https://ecdc.europa.eu/en/publications-data/sixth-external-quality-assessment-scheme-listeria-monocytogenes-typing

 

The use of evidence in decision making during public health emergencies

Date of publication: 26 July 2019

https://ecdc.europa.eu/en/publications-data/use-evidence-decision-making-during-public-health-emergencies

 

Investigation and public health management of people with possible Ebola virus disease infection

Date of publication: 19 July 2019

https://ecdc.europa.eu/en/publications-data/investigation-and-public-health-management-people-possible-ebola-virus-disease

 

Syphilis and congenital syphilis in Europe - A review of epidemiological trends (2007–2018) and options for response

Date of publication: 12 July 2019

https://ecdc.europa.eu/en/publications-data/syphilis-and-congenital-syphilis-europe-review-epidemiological-trends-2007-2018

**Surveillance reports**

 

Monthly measles and rubella monitoring report, September 2019

Date of publication: 13 September 2019

https://ecdc.europa.eu/en/publications-data/monthly-measles-and-rubella-monitoring-report-september-2019

 

Influenza virus characterisation, July 2019

Date of publication: 3 September 2019

https://ecdc.europa.eu/en/publications-data/influenza-virus-characterisation-july-2019

 

Monthly measles and rubella monitoring report, August 2019

Date of publication: 9 August 2019

https://ecdc.europa.eu/en/publications-data/monthly-measles-and-rubella-monitoring-report-august-2019

 

Monthly measles and rubella monitoring report, July 2019

Date of publication: 19 July 2019

https://ecdc.europa.eu/en/publications-data/monthly-measles-and-rubella-monitoring-report-july-2019

 

Influenza virus characterisation, June 2019

Date of publication: 15 July 2019

https://ecdc.europa.eu/en/publications-data/influenza-virus-characterisation-summary-europe-june-2019

 

**Mission reports**

Country visit to Estonia to discuss policies relating to antimicrobial resistance

Date of publication: 16 September 2019

https://ecdc.europa.eu/en/publications-data/ecdc-and-european-commission-country-visit-estonia-discuss-policies-relating

 

**Annual Epidemiological Report series on communicable diseases in Europe**

****

**Published in July:**

****

Chapter on tick-borne encephalitis for 2017

https://ecdc.europa.eu/en/publications-data/tick-borne-encephalitis-annual-epidemiological-report-2017

 

Chapter on Zika virus disease for 2017

https://ecdc.europa.eu/en/publications-data/zika-virus-disease-annual-epidemiological-report-2017

 

Chapter on malaria for 2017

https://ecdc.europa.eu/en/publications-data/malaria-annual-epidemiological-report-2017

 

Chapter on Ebola and Marburg fevers for 2017

https://ecdc.europa.eu/en/publications-data/ebola-and-marburg-fevers-annual-epidemiological-report-2017

 

Chapter on hantavirus for 2017

https://ecdc.europa.eu/en/publications-data/hantavirus-infection-annual-epidemiological-report-2017

 

Chapter on tularaemia for 2017

https://ecdc.europa.eu/en/publications-data/tularaemia-annual-epidemiological-report-2017

 

Chapter on syphilis for 2017

https://ecdc.europa.eu/en/publications-data/syphilis-annual-epidemiological-report-2017

 

**Corporate publication**

Achievements, challenges and major outputs 2018: Highlights from the Annual Report of the Director

Date of publication: 28 August 2019

https://ecdc.europa.eu/en/publications-data/achievements-challenges-and-major-outputs-2018-highlights-annual-report-director

 

**ECDC communicable disease threats report**

Date of publication: every Friday

https://ecdc.europa.eu/en/threats-and-outbreaks/reports-and-data/weekly-threats

**

**Scientific peer-reviewed publications**

Publications by ECDC staff in scientific journals

Updated monthly

https://ecdc.europa.eu/en/publications-data?f%5b0%5d=output_types%3A1247

